# Identification and Phenotypic Characterization of Hsp90 Phosphorylation Sites That Modulate Virulence Traits in the Major Human Fungal Pathogen *Candida albicans*

**DOI:** 10.3389/fcimb.2021.637836

**Published:** 2021-08-27

**Authors:** Leenah Alaalm, Julia L. Crunden, Mark Butcher, Ulrike Obst, Ryann Whealy, Carolyn E. Williamson, Heath E. O’Brien, Christiane Schaffitzel, Gordon Ramage, James Spencer, Stephanie Diezmann

**Affiliations:** ^1^Department of Biology & Biochemistry, University of Bath, Bath, United Kingdom; ^2^School of Cellular and Molecular Medicine, University of Bristol, Bristol, United Kingdom; ^3^School of Medicine, Dentistry and Nursing, University of Glasgow, Glasgow, United Kingdom; ^4^MRC Centre for Neuropsychiatric Genetics & Genomics, Division of Psychological Medicine & Clinical Neurosciences, Cardiff University, Cardiff, United Kingdom; ^5^School of Biochemistry, University of Bristol, Bristol, United Kingdom

**Keywords:** fungal virulence, phospho-switch, Hsp90, thermotolerance, drug response, morphogenesis, *Candida albicans*

## Abstract

The highly conserved, ubiquitous molecular chaperone Hsp90 is a key regulator of cellular proteostasis and environmental stress responses. In human pathogenic fungi, which kill more than 1.6 million patients each year worldwide, Hsp90 governs cellular morphogenesis, drug resistance, and virulence. Yet, our understanding of the regulatory mechanisms governing fungal Hsp90 function remains sparse. Post-translational modifications are powerful components of nature’s toolbox to regulate protein abundance and function. Phosphorylation in particular is critical in many cellular signaling pathways and errant phosphorylation can have dire consequences for the cell. In the case of Hsp90, phosphorylation affects its stability and governs its interactions with co-chaperones and clients. Thereby modulating the cell’s ability to cope with environmental stress. *Candida albicans*, one of the leading human fungal pathogens, causes ~750,000 life-threatening invasive infections worldwide with unacceptably high mortality rates. Yet, it remains unknown if and how Hsp90 phosphorylation affects *C. albicans* virulence traits. Here, we show that phosphorylation of Hsp90 is critical for expression of virulence traits. We combined proteomics, molecular evolution analyses and structural modeling with molecular biology to characterize the role of Hsp90 phosphorylation in this non-model pathogen. We demonstrated that phosphorylation negatively affects key virulence traits, such as the thermal stress response, morphogenesis, and drug susceptibility. Our results provide the first record of a specific Hsp90 phosphorylation site acting as modulator of fungal virulence. Post-translational modifications of Hsp90 could prove valuable in future exploitations as antifungal drug targets.

## Introduction

Fungi kill as many patients as tuberculosis and about three times as many as malaria each year (Bongomin et al., 2017). One of the most deadly fungal pathogens, *Candida albicans* causes ~750,000 cases of invasive life-threatening bloodstream infections in immunocompromised patients worldwide each year (Bongomin et al., 2017), with mortality rates approaching 75% (Brown et al., 2012). In addition to high burdens on human life, fungal infections put a significant strain on health care costs. The United States alone spent $4.5 billion on 75,055 hospitalizations necessitated by fungal disease over ten years (Benedict et al., 2018) while English NHS trusts spend annually approximately £90 million on antifungal drugs (Whitney et al., 2018). This already dire situation is further exacerbated by the ever-growing number of patients at risk of contracting invasive fungal infections. It is thus imperative to understand the biological principles underpinning fungal virulence to identify points of fragility suitable for therapeutic targeting.

Diverse aspects of fungal virulence are controlled by heat shock protein 90 (Hsp90), a ubiquitous and highly conserved regulator of the cellular protein homeostasis. Hsp90 regulates cell morphology, drug resistance, and virulence in *C. albicans* (Singh et al., 2009) and other leading fungal pathogens of humans such *Cryptococcus neoformans* (Cordeiro et al., 2015), which causes brain infections, and *Aspergillus fumigatus* (Lamoth et al., 2012) which targets the lungs, as well as the dermatophyte *Trichophyton rubrum* (Jacob et al., 2015), and *Sporothrix schenckii* (Rodriguez-Caban et al., 2011), the causative agents of cutaneous infections. Hsp90’s role in virulence and development is best understood in *C. albicans*, where pharmacological inhibition or genetic reduction of Hsp90 result in reduced drug resistance in planktonic cells (Singh et al., 2009) and biofilms (Robbins et al., 2011), switching to filamentous growth (Shapiro et al., 2009), induction of same-sex mating (Guan et al., 2019), and delayed cell cycle progression (Senn et al., 2012). Although attractive as a drug target for its role in fungal virulence, mice infected with *C. albicans* and treated with Hsp90 inhibitors developed as anti-cancer therapeutics together with antifungal drugs, suffer severe host toxicity (Cowen et al., 2009). Targeting fungal Hsp90 but not host Hsp90 in the context of candidemia is challenging due to high sequence conservation (Swoboda et al., 1995). Species-specific targeting of distinct Hsp90 features, such as the recently identified *C. albicans*-specific binding mode of the nucleotide-binding pocket (Whitesell et al., 2019), could provide a novel avenue to address this issue. Alternatively, understanding fungal-specific regulatory mechanisms of Hsp90 could provide a therapeutic approach that would consequently be of biological and clinical relevance.

Post-translational modifications (PTMs) play a crucial role in regulating Hsp90. Their impact on chaperone activity, directionality of the chaperone cycle, and binding to co-chaperones has been extensively studied in mammalian cells and the model eukaryote *Saccharomyces cerevisiae* (Mollapour and Neckers, 2012). However, examples of Hsp90 PTMs in pathogenic fungi are limited to the observations that concurrent acetylation of two lysine residues, K30 and K271, resulted in increased susceptibility to Hsp90 inhibition, morphogenetic alterations, and impaired macrophage pyroptosis in *C. albicans* (Li et al., 2016), as well as attenuated virulence in *A. fumigatus* (Lamoth et al., 2014).

Given the importance of kinases as drug targets (Cohen, 2002) and accumulating experimental evidence of phosphorylation regulating most Hsp90 activities, this type of PTM is of particular interest. Phosphorylation of mammalian and *S. cerevisiae* Hsp90 negatively affects the chaperone cycle, attenuates interactions with clients and co-chaperones, induces conformational switching, and increases sensitivity to Hsp90 inhibitors (Sima and Richter, 2018). The ubiquitous, tetrameric casein kinase 2 (CK2) is one of several kinases targeting Hsp90 (Lees-Miller, 1989; Mollapour and Neckers, 2012). CK2, which is best known for its central role in the regulation of cell proliferation and DNA damage repair (Filhol and Cochet, 2009), is a ubiquitous, constantly active, hetero-tetrameric Ser/Thr kinase. In *S. cerevisiae*, a close relative of *C. albicans*, CK2 is composed of two catalytic subunits α and α’, encoded by *CKA1* and *CKA2*, and two regulatory subunits β and β’, encoded by *CKB1* and *CKB2*. The enzyme’s four active forms, αα’ββ’, α_2_ββ’, α’_2_ββ’, and α’, phosphorylate different substrates (Domańska et al., 2005). In *Candida albicans*, homologous genes have been identified and deletion of *CKA2* (α’), which has 65% sequence identity with the *S. cerevisiae* homolog, results in resistance to the broadly deployed antifungal fluconazole (Bruno and Mitchell, 2005). In both yeast species, mutations in *CKA1* and *CKA2* are synthetically lethal (Padmanabha et al., 1990; Bruno and Mitchell, 2005).

In Hsp90, CK2 phosphorylates the highly conserved T36 in mammalian cells and T22 in *S. cerevisiae* (Mollapour et al., 2011). Phosphorylation of T22 or T36 reduces kinase client stability and co-chaperone binding of Hsp90 in *S. cerevisiae* and mammalian cells. While at least 14 CK2 phosphorylation sites have been identified in human Hsp90, phosphorylation patterns in non-model organisms remain enigmatic. In *C. albicans*, probing purified Hsp90 with phospho-serine and phospho-threonine specific antibodies revealed severely reduced serine phosphorylation in a mutant lacking the *CKB1* (β) subunit of CK2 (Diezmann et al., 2012). Yet, specific Hsp90 serine residues targeted by CK2 and the functional consequences of Hsp90 phosphorylation in *C. albicans* have remained elusive so far.

Here we combined proteomics with *Candida* molecular genetics to identify and characterize CK2 phosphorylation sites in Hsp90. Mass spectrometric analyses of purified Hsp90 revealed three novel CK2 target sites. One of these was selected for further characterization together with the T22/36 residue, which was not detected as differentially phosphorylated but was selected based on evolutionary conservation. This study demonstrates that phosphorylation of the newly identified Hsp90 residue S530 blocks expression of virulence-related traits in culture, such as cellular morphogenesis and survival of thermal stress. Probing the conserved T22/36 residue revealed that any alteration of this residue leads to phenotypes indicative of loss of Hsp90 function. Our results emphasize the importance of Hsp90 phospho-regulation for fungal virulence traits and provide a potential site for future species-specific therapeutic targeting.

## Matherials and Methods

### Strains, Strain Construction and Culture Conditions

All strains used in this study are listed in **Table S1**. A detailed description of strain and plasmid construction can be found in the Supplementary Information together with **Tables S2** and **S3**, which list the necessary primers. In preparation for experiments, strains were grown for 16-18 hours in YPM (1% yeast extract, 2% peptone, 2% maltose) at 30°C while shaking at 200 rpm, unless specified otherwise. To repress the HSP90 wild-type allele, strains were grown in YPD (1% yeast extract, 2% peptone, 2% dextrose) at the temperatures specified. For long-term storage, strains were maintained at -80°C in 25% glycerol.

### Identification of the S530 Phosphorylation Site

The S530 phosphorylation site in Hsp90 was identified by mass spectrometric analysis of purified Hsp90 in the Proteomics Core Facility at EMBL Heidelberg, Germany. To purify Hsp90 from CK2 deletion mutants (YSD692, YSD675, YSD694, YSD696) and the corresponding wild type (YSD673), strains were grown to mid-log phase at 30°C in 200 ml volumes in YPD at 30°C. Whole cell proteins were extracted as described below and the C-terminally TAP-tagged chaperone was pulled down using IgG agarose beads from lysate containing 20 mg of protein (see below). The protein was eluted in 100 µl 1x sample buffer and heated to 95°C for 10 minutes prior to loading 30 µl onto a 10% Tris-glycine SDS-PAGE gel for separation. Coomassie-stained gels were imaged and the bands corresponding to Hsp90 were excised and processed by in-gel digestion (Pereira et al., 2019). Hsp90’s phosphorylation sites were identified using Glu-C and trypsin digestion and liquid chromatography-mass spectrometry on a QExactive plus mass spectrometer (Thermo Fisher).

### Structural Modeling

A model of the full-length *C. albicans* Hsp90 monomer was generated based on the *S. cerevisiae* Hsp90-Sba1 crystal structure (Ali et al., 2006) (PDB #2cg9) using the Phyre^2^ engine (Mezulis et al., 2015). The model was aligned to the *S. cerevisiae* sequence and visualized using the PyMOL Molecular Graphics System (Schrödinger, 2010). Models of Hsp90 phosphorylated at positions 25 or 530 were generated in Coot (Emsley et al., 2010) and electrostatic potential surfaces generated using CCP4mg molecular-graphics software (McNicholas et al., 2011).

### Ancestral Character State Reconstruction

Sequences of 240 fungal taxa were obtained from the Ensembl Genomes database for fungi and aligned with the murine homolog Hsp90ab1 (**Table S4**) in AliView version 1.26 (Larsson, 2014) using the integrated default alignment program MUSCLE (Edgar, 2004). A Neighbor Joining tree was built in MEGA version 10.1.8 (Kumar et al., 2018) and loaded into Mesquite version 3.61 (Maddison and Maddison) for ancestral character state reconstruction using the parsimony criterion.

### Western Blotting and Co-Immunoprecipitations

To determine Hsp90 stability in the wild-type, *MAL2-HSP90* control and *hsp90* mutant strains, stationary phase cells grown in YPM were inoculated into YPD or YPM at an OD_600_ of 0.2 and incubated until they reached a log-phase OD_600_ between 1.3 and 1.9 (approximately 4.5 hours). 50 ml of cell culture were pelleted, snap frozen in liquid nitrogen, and stored overnight at -20°C. Pellets were thawed on ice, washed twice with 1x PBS and resuspended in 250 µl lysis buffer (50 mM HEPES pH 7.5, 150 mM NaCl, 5 mM EDTA, 1% Triton X100, 1 cOmplete EDTA-free protease inhibitor tablet, 1 mM PMSF). Cells were transferred to Precellys 2 ml Tough Micro-organism Lysing Kit tubes (VK05) and lysed using a Precellys Evolution tissue homogenizer. Samples were processed in 3 cycles, each consisting of bead beating at 6,000 rpm for 1 minute, pause for 30 seconds, bead beating at 6,000 rpm for 1 minute. Tubes were rested on ice for at least 1 minute between each cycle. Lysates were cleared twice by centrifugation at 20,000 xg at 4°C for 30 minutes, and 5 minutes. Supernatants were transferred to microfuge tubes and refrigerated in preparation for sample preparation and gel loading. The lysates’ whole cell protein concentrations were determined with a Quick Start Bradford Protein Assay (Bio-Rad) and calibrated against a BSA standard curve. Samples were diluted to 0.1 µg/µl protein in sample buffer (70 mM Tris-HCl pH 6.8, 11.1% glycerol, 1.1% SDS, 0.005% bromophenol blue, 10% 2-mercaptoethanol, all final concentrations), heated at 95°C for 10 minutes and either cooled on ice prior to loading or stored at -20°C.

As loading controls, 100 µl per sample were loaded onto a 10% SDS-PAGE gel and separated for 20 minutes at 90 volts, followed by 100 minutes at 120 volts. To visualize proteins, gels were stained with SimplyBlue SafeStain (Invitrogen) according to the manufacturer’s instructions and imaged by scanning.

For immunoblotting of *C. albicans* Hsp90, 15 µl were loaded on a 10% SDS-PAGE gel and separated for 20 minutes at 90 volts, followed by 100 minutes at 120 volts. Proteins were wet-transferred onto a PVDF membrane (Bio-Rad) for 135 minutes at 300 mA in a Mini-PROTEAN Tetra Vertical Electrophoresis Cell filled with transfer-buffer (2 M glycine, 0.25 M Tris-base, 20% methanol). To block free sites, membranes were treated with PBST (1x PBS, 0.1% Tween 20) with 0.1% non-fat milk overnight at 4°C. The blots were probed for 1 hour at room temperature with α-CaHsp90 (Burt et al., 2003) diluted 1:10,000 in PBST with 0.1% milk and washed with 1x PBST prior to probing with α-rabbit antibody-HRP conjugate (BioRad #1705046) for 15 minutes at room temperature. Lastly, blots were washed with 200 ml PBST using the SNAP i.d. 2.0 Protein Detection System (Merck Millipore), incubated with Clarity Western ECL (BioRad), and imaged on the Syngene G:Box transilluminator.

To purify Hsp90 for mass-spectrometric analysis and to investigate the effect of Hsp90 phospho-mutant alleles on co-chaperone binding, 200 ml of cells were grown as described above, washed twice in 1x PBS and resuspended in 2 ml co-IP lysis buffer (50 mM Tris-HCl pH 7.5, 1% Nonidet P 40 Substitute (Sigma #74385), 0.25% deoxycholate Na, 150 mM NaCl, 5 mM EDTA, 1 mM Na_2_MoO_4_•2H_2_O, PhosSTOP tablet, cOmplete EDTA-free protease inhibitor tablet, 1 mM PMSF, 1 mM Na_3_VO_4_, and 10 mM NaF). Cells were transferred to 15 ml Precellys sample tubes (VWR BERT KT03961-1-4061), mixed with 1 ml acid-washed glass beads (~400 µm) and mechanically disrupted using a Precellys Tissue Homogeniser. Tubes were agitated three times for one minute with one minute on ice in between. The lysate was cleared by centrifugation at 20,000 *x*g twice for 5 minutes at 4°C and protein concentrations were determined using the Bradford assay described above. 7.5 mg of protein were mixed with 100 µl of IgG or α-HA agarose, depending on the strain’s genotype, in co-IP washing buffer (50 mM Tris-HCl pH 7.5, 1% Nonidet P 40 substitute, 0.25% deoxycholate Na, 150 mM NaCl, 5 mM EDTA, 1 mM Na_2_MoO_4_•2H_2_O, 1 mM PMSF, 1 mM Na_3_VO_4_, and 10 mM NaF) and gently rotated for 2 hours at 4°C. Following precipitation, the agarose beads were washed carefully 3-5 times with 1 ml co-IP washing buffer before being mixed with 100 µl 1x sample buffer (6x sample buffer: 0.35 M Tris HCl, 10% (w/w) SDS, 36% glycerol, 5% 2-mercaptoethanol and 0.012% bromophenol blue). Prior to loading, samples were heated at 95°C for 10 minutes and cooled on ice ready for loading or stored at -80°C. Proteins were separated at 120 volts on size-appropriate SDS-PAGE gels.

To visualize epitope-tagged co-chaperones, proteins were wet-transferred onto PVDF membranes for 16 hours at 20 volts in a Mini-PROTEAN Tetra Vertical Electrophoresis Cell filled with transfer-buffer (2 M glycine, 0.25 M Tris-base, 20% methanol). Membranes were blocked with PBST and 5% milk for at least 1 hour at room temperature before being probed with primary and secondary antibodies. To visualize TAP-tagged Sti1, membranes were treated with α-TAP antibody (Open Biosystems #CAB1001). Membranes carrying HA-tagged Cdc37, Aha1 or Sba1 were exposed to α-HA antibody (Invitrogen #71-5500). Both primary antibodies were diluted 1:5,000 in PBST with 0.2% milk and subsequently conjugated with α-rabbit antibody-HRP conjugate (BioRad Immun-Star Goat Anti-Rabbit (GAR)-HRP Conjugate #1705046) diluted 1:5,000. Membranes were incubated in antibody solutions for 15 minutes each and washed at least three times with 1x PBST using the SNAP i.d. 2.0 Protein Detection System. Lastly, proteins were visualized with Clarity Western ECL (BioRad).

### Temperature Growth Profiles

To assess susceptibility to thermal stress, optical densities of stationary phase cultures were measured at 600 nm and adjusted to 0.1 in 1x PBS and serially diluted two-fold. 4 µl of cell suspension were then spotted onto solid YPD or YPM and plates were incubated at the indicated temperature for 48 hours. Plates were photographed and analyzed visually.

### Cellular Morphogenesis Analysis

Stationary phase cells grown in YPM at 30°C were diluted to an optical density of 0.2 in YPD or YPM and grown for 16 hours at 30°C while shaking at 200 rpm. In preparation for microscopy, cells were washed and diluted with 1x PBS to a cell density appropriate for microscopy. Images were collected using the DIC settings on a Nikon Eclipse E8000 microscope with a Nikon Digital Sight DS-5M camera. To calculate the morphology index (MI) (Mersondavies and Odds, 1989), a total of 100 cells per strain were randomly selected and their width at the septal junction as well as cell length and maximum diameter measured using ImageJ (Schneider et al., 2012). The MI for each cell was then presented in scatter plots.

### Drug Susceptibility Assays

Strains were grown to stationary phase at 30°C in YPM while shaking at 200 rpm and their optical densities were measured at 600 nm and adjusted to ~10^3^ cells/100 µl. Fluconazole was diluted in water to a starting concentration of 512 µg/ml and 20 µl of drug were mixed with 180 µl YPD or YPM in a 96-well flat bottom dish. The drug was then diluted two-fold in YPD or YPM to cover a gradient ranging from 256 µg/ml to 4 µg/ml. 100 µl of cell suspension were added to each well and plates were incubated for 48 hours at 25°C and optical densities recorded at 595 nm. A 5 mM radicicol stock solution (DMSO) was diluted to 50 µg/ml in YPM or YPD. The drug was diluted in either media to the following concentrations: 1, 2, 3, 4, 5, 7.5, 10, 15, 20, and 25 µg/ml and strains inoculated as above. To account for the effects of DMSO alone, strains were also inoculated in 0.0275% DMSO. Plates were incubated for 48 hours at 25°C in the dark and optical densities recorded at 595 nm. Optical density values were normalized to the no treatment column and visualized using Java TreeView version 1.1.6r4 (Saldanha, 2004).

### Colony Morphology Screen

Wild-type, control and mutant strains were grown overnight in YPM at 30°C while shaking at 200 rpm. Stationary cultures were then diluted to an optical density of 0.02 at 600 nm and 5 µl of cell suspension spotted onto RPMI, Spider, and synthetic defined media with and without Calcofluor White or Congo Red (**Table S5**). After incubation at 30°C for 48 hours colonies were visually scored for appearance as ‘smooth’, ‘rough’, ‘wrinkly’, or ‘wrinkly with hyphal edge’ and photographed.

### Biofilm Analyses

In preparation for biofilm analyses, C. albicans strains were cultured overnight in YPD at 30°C while shaking at 150 rpm. Stationary phase cells were centrifuged at 4,400 rpm for 5 minutes, washed with 1x PBS and counted using a Neubauer hemocytometer. To induce biofilm formation, cells were diluted to 10^6^ cells/ml in RPMI (Sigma-Aldrich/Merck # R7755) and incubated at 37°C for 24 hours either in flat-bottom 96-well dishes to quantify metabolic activity or on Thermanox™ coverslips to assess structural integrity via scanning electron microscopy. Metabolic activity, serving as a proxy for cell viability, was assessed by metabolic reduction of resazurin sodium salt (Sigma-Aldrich/Merck #R7017) (Fai and Grant, 2009; Chadha and Kale, 2015). For the assay, 0.1 g of resazurin was dissolved in 10 ml 1x PBS and filter-sterilized. Biofilm wells were washed twice with 1x PBS and filled with 100 µl of a 1:1000 dilution of resazurin in RPMI. Plates were incubated at 37°C for 25 minutes and emission was measured at 590 nm following excitation at 544 nm (Delpech et al., 2018). To visualize biofilms grown on coverslips, they were washed once with 1x PBS and fixed overnight at 4°C in 500 µl of 0.15% w/v Alcian Blue dissolved in 2% para-formaldehyde, 2% glutaraldehyde, and 0.15 M sodium cacodylate. Samples were imaged as described previously (Erlandsen et al., 2016).

### Statistical Analyses

All analyses were carried out in R Version 3.6.0 and plotted using ggplot2. To assess the effect of the Hsp90 mutant alleles on cellular morphology, the MIs for samples grown in YPD and YPM were compared with each other using the Mann-Whitney U test. Statistical differences between biofilm metabolic activity were assessed by a linear model of strain versus optical density using Ordinary Least Squares regression. P-values were calculated from F statistics comparing the mutant strains to the maltose control.

## Results

### Mapping, Modeling, and Evolutionary History of CK2 Hsp90 Phosphorylation Sites

To map CK2 phosphorylation sites in *C. albicans* Hsp90 *in situ*, the wild-type strain and four mutants, each lacking one CK2 subunit, were grown in rich media at 30°C upon which epitope tagged Hsp90 was purified and analyzed by mass spectrometry. Without further enrichment, Hsp90 sequence coverage in trypsin digests was 70% or higher and ranged between 40-45% in endoproteinase Glu-C digests. Three serine phosphorylation sites were identified using mass spectrometry (**Table 1**). S279 was only phosphorylated in the *cka1Δ/Δ* mutant. S294 was phosphorylated in the wild type and all four CK2 mutants. S530 was phosphorylated in the wild type, the *cka1Δ/Δ* and the *ckb1Δ/Δ* mutants, but not the *cka2Δ/Δ* and *ckb2Δ/Δ* mutants. Since S530 was detected with the highest confidence (99-100%), we decided to focus on this residue. Lack of phosphorylation of S530 in the *cka2Δ/Δ* mutant suggests this residue could be relevant for the increased fluconazole resistance previously observed in the *cka2* mutant (Bruno and Mitchell, 2005). Furthermore, drug resistance is abolished when Hsp90 levels are perturbed either genetically or pharmacologically (Cowen and Lindquist, 2005).

**Table 1:** Hsp90 phospho-peptides identified by mass spectrometry in the wild type (WT) and the CK2 mutants.

Strain Name (ID)	CK2 subunit	Site	Peptide sequence (after Glu-C and trypsin digestion)	Score peptide	Confidence in site (%)
SN95 (YSD89)		S294	K.SI**S**NDWEDPLAVK.H	53	90.46
		S294	K.SI**S**NDWEDPLAVKHFSVEGQLEFR.A	35	73.23
		S530	K.LVDITKDFELEE**S**DEEKAAR.E	80	100.0
cka1 (YSD557)	α	S279	R.NP**S**DITQDEYNAFYK.S	38	99.36
		S294	K.SI**S**NDWEDPLAVK.H	59	50.0
		S294	K.SI**S**NDWEDPLAVKHFSVEGQLEFR.A	39	66.5
		S530	K.LVDITKDFELEE**S**DEEKAAR.E	80	100.0
		S530	K.LVDITKDFELEE**S**DEEKAAR.E	36	99.93
cka2 (YSD623)	α’	S294	K.SI**S**NDWEDPLAVK.H	63	92.26
ckb1 (YSD628)	β	S294	K.SI**S**NDWEDPLAVK.H	71	94.30
		S530	K.LVDITKDFELEE**S**DEEKAAR.E	47	99.93
ckb2 (YSD634)	β’	S294	K.SI**S**NDWEDPLAVK.H	34	50.0

Phosphorylated serine residues are highlighted in bold red.

We thus hypothesized that phosphorylation of S530 blocks Hsp90 function, thereby reducing resistance to antifungal drugs. The acquisition of a PTM that reduces drug resistance appears counter-intuitive, but the trade-off theory of optimal virulence predicts selective pressure to evolve reduced virulence in cases where the host and the pathogen’s fitness are aligned (Ewald, 1983). As a commensal, *C. albicans*’ primary niche are the mucosal surfaces of the oral cavity and the GI tract. To test this, we assessed the effect of Hsp90^S530^ phosphorylation on diverse virulence traits, including high temperature growth and morphogenesis.

**Figure 1:**
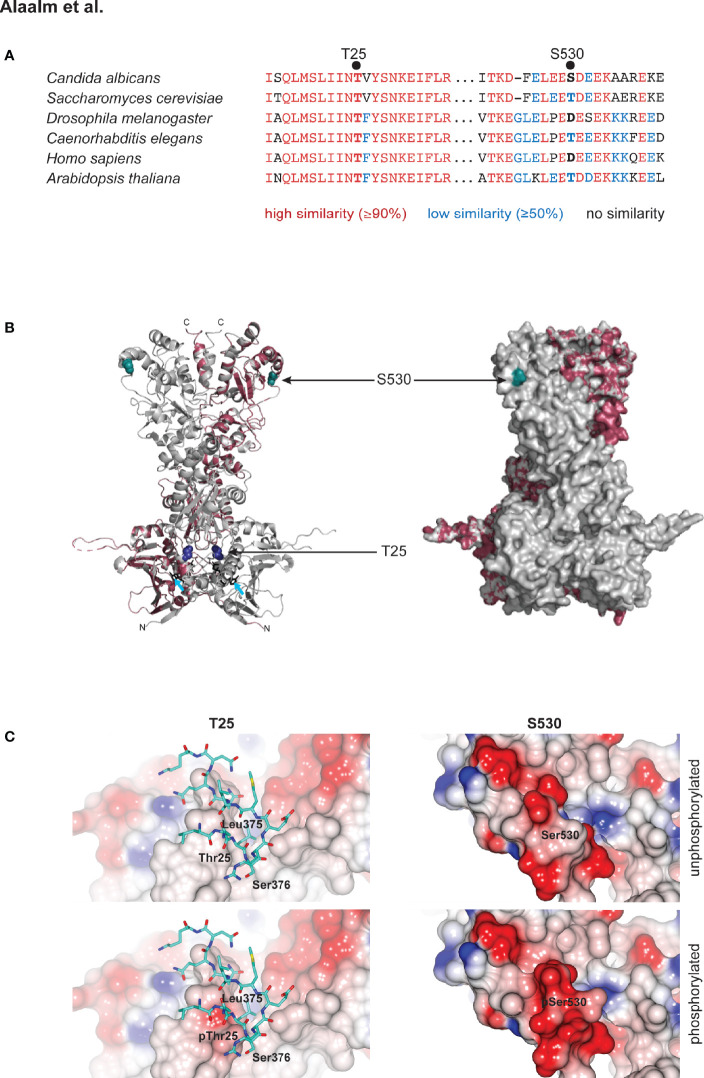
Structural modeling of CK2 phosphorylation sites in Hsp90. **(A)** Alignment of eukaryotic Hsp90 protein sequences, comparing two fungal sequences to three animal and one plant protein. T25 resides in a highly conserved (red) region of the N-terminal domain of Hsp90. S530 is located in a divergent (blue) region of the Hsp90 C-terminus. **(B)** *C*. *albicans* Hsp90 (purple) modeled on the full-length crystal structure of *S. cerevisiae* Hsp90 (grey). T25 (blue) and S530 (teal) are visualized on the homology model (left) and the surface model (right). Azure arrows point to bound ATP in the homology model. **(C)** Electrostatic surfaces of Hsp90 in the vicinity of T25 (left) and S530 (right). Surface of one Hsp90 monomer is colored by electrostatic potential from -0.5 V (red) to +0.5 V (blue). In left hand panels, residues 373 – 384 of the second monomer of the Hsp90 dimer are rendered as sticks (carbon atoms cyan, other atom colors as standard). Upper panels, wild type Hsp90. Lower panels, Hsp90 with T25 or S530 phosphorylated.

Although elusive in our mass spectrometric analyses, we decided to also characterize the *C. albicans* homolog of the conserved CK2 target T22/36, which is Hsp90^T25^, called T25 henceforth. *S. cerevisiae* T22 was initially identified as critical for the survival of elevated temperatures (Borkovich et al., 1989). T25 resides in a highly conserved region of Hsp90’s N-terminus, which contains the ATP binding site that is the target of the Hsp90 inhibitors geldanamycin and radicicol (Pearl and Prodromou, 2006). S530 is located in a region of high sequence divergence, at the very beginning of the dimerizing C-terminus (**Figure 1A**) (Girstmair et al., 2019). To elucidate the relationship of these two residues within the Hsp90 molecule, we modeled the full-length *C. albicans* Hsp90 monomer on the *S. cerevisiae* crystal structure (Ali et al., 2006) (**Figure 1B**). A total of 609 residues (94% coverage) were aligned with 100% confidence, sharing 89% identity between both yeast species. The homology model revealed that both residues are located in loop regions without secondary structural elements (**Figure 1B**). T25 resides in close proximity to the ATP binding pocket, while S530 is located at the very beginning of the protein’s C-terminus (Girstmair et al., 2019). The surface model shows that T25, although not a hydrophobic amino acid, is not solvent exposed, while S530 is (**Figure 1B**). These observations are in keeping with molecular evolution theory, which predicts that decreased solvent accessibility results in fewer amino acid polymorphisms (Bustamante et al., 2000) and thus higher sequence conservation. *In silico* modeling of the effects of T25 or S530 phosphorylation on surface charge indicates that T25 phosphorylation introduces charge to what is a predominantly hydrophobic and functionally important interface with residues 373 – 384 in the middle domain of the opposing subunit in the Hsp90 dimer, while S530 phosphorylation increases the charge of what is already an acidic region of the protein surface (**Figure 1C**).

Given the divergent nature of the Hsp90 region S530 resides in (**Figure 1A**), we investigated the residue’s evolutionary history within the fungal kingdom, asking if the *C. albicans* serine allele presented a unique case or if multiple transitions within the fungi could be detected. Aligning 659 amino acids of 240 fungal Hsp90 sequences, representing nine sub-phyla, with the murine homolog Hsp90ab1 revealed serine to be a derived allele originating at least eleven times independently within the Dikarya, which encompass the Ascomycota and the Basidiomycota, and represent ~96% of known fungal species (Blackwell, 2011) (**Figures S1**, **S2** and **Table S4**). Basal fungi, such as the Chytridiomycota, share an aspartic acid allele with animal cytosolic Hsp90. Threonine originated in the last common ancestor of the Dikarya and threonine to serine transitions were detected in five of the six sub-phyla. Serine alleles were detected in species considered plant pathogens (*Ustilago hordei*, *Puccinia* complex, *Aspergillus niger, Ashbya gossypii*), species associated with trees (*Neolecta irregularis*), fermentative yeast (*Nadsonia fulvescens*), methylotrophic yeasts *(Komagataella phaffi*, *K. pastoris*, *Ogataea parapolymorpha*, *O. polymorpha*), halo-tolerant species (*Debaryomyces hansenii, Zygosaccharomyces rouxii*) and the opportunistic pathogens (*Meyerozyma guilliermondii*, *Malassezia globosa*, *Candida parapsilosis*). No serine alleles were detected amongst the 82 Agaromycotina, which include the human pathogen *Cryptococcus neoformans* as well as mushrooms and jelly fungi. In summary, the serine allele is rare; it is present in less than 5% of fungi. Serine occurs in species that are capable of causing disease in plants, thrive in extreme environments, and exist as commensals of the human body where they can cause opportunistic infections in responses to changes in the host’s immune status, like *C. albicans*.

### Hsp90 Phosphorylation Negatively Affects Protein Stability and Co-C34haperone Binding

To investigate the effects of phosphorylation of T25 and S530 and to accommodate the ‘obligate diploid’ nature of *C. albicans*, we generated strains expressing one mutant *hsp90* allele, where the relevant residue was replaced with either a phosphomimetic glutamic acid (E) or a non-phosphorylatable alanine (A) while the remaining *HSP90* wild-type allele was placed under the control of the maltose-inducible promoter *MAL2*p. Consequently, strains grown in dextrose solely expressed the mutant allele, thereby revealing the effects of phosphorylation. Growth in maltose resulted in expression of the wild-type allele, serving as control and for strain maintenance. Non-phosphorylatable and phosphomimetic alleles were created for both residues (T25A, T25E, S530A, S530E) and compared to the wild type and the *MAL2-HSP90* promoter control, that carries two *HSP90* wild-type alleles, one of which is under the control of the *MAL2* promoter.

**Figure 2:**
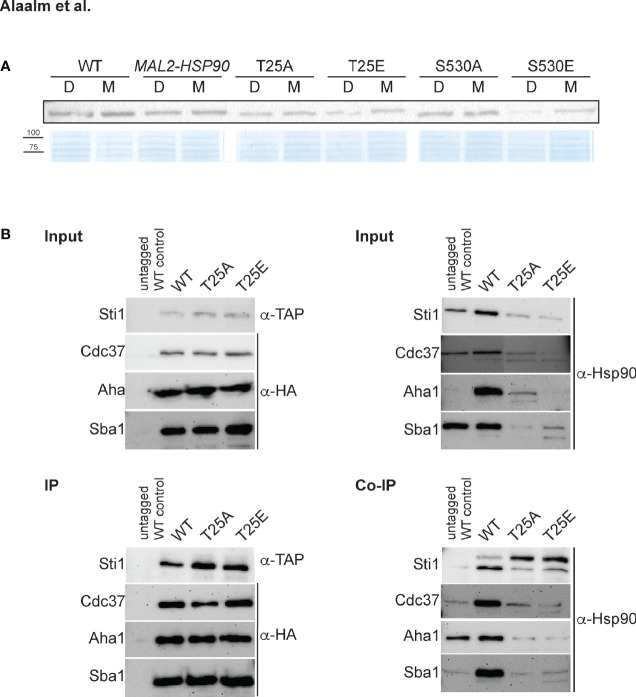
The *C. albicans* Hsp90 co-chaperone machinery is regulated by phosphorylation. **(A)** One of two Western blots probed for presence of Hsp90 in *C. albicans* wild-type (WT), promoter control (*MAL2-HSP90*) and T25 and S530 mutant strains (top). Strains were grown in media containing either maltose (M) or dextrose (D) to regulate expression of mutant *hsp90 via the* maltose-inducible promoter. A Coomassie-stained SDS gel validates equal loading of protein samples (bottom). **(B)** TAP-tagged Sti1 and HA-tagged co-chaperones Cdc37, Aha1, and Sba1 were co-immunoprecipitated with Hsp90 in the wild type (WT) and the T25 mutants. The input was first probed for presence of co-chaperones and Hsp90 prior to immunoprecipitation with IgG or α-HA agarose targeting the co-chaperones. Levels of co-chaperones were then assessed in the immunoprecipitate (IP) using α-TAP and α-HA antibodies. To screen for co-immunoprecipitated Hsp90 (Co-IP), the membrane was probed with an Hsp90-specific antibody.

To test if phosphorylation affects Hsp90 stability, strains were grown in dextrose or maltose and cell extracts probed for Hsp90 using an antibody that recognizes amino acid residues 693-702 in the *C. albicans* Hsp90 C-terminus (Burt et al., 2003). Replacing T25 or S530 with the phosphomimetic residue (E) reduced Hsp90 levels in cells grown in dextrose when compared to the *MAL2-HSP90* control while protein levels remained comparable in the strains carrying the non-phosphorylatable alanine allele (A) (**Figure 2A**). This suggests that phosphorylation of T25 or S530 destabilizes Hsp90. Alternatively, introduction of a glutamic acid residue could destabilize the Hsp90 mutant form.

Given the importance of T22/T36 for co-chaperone binding in *S. cerevisiae* and mammalian cells (Mollapour et al., 2011), we aimed to determine if the homologous T25 residue in *C. albicans* plays a similar role. Cell lysates from strains with TAP or HA tagged co-chaperones were immunoprecipitated with IgG or α-HA agarose and probed for presence of co-chaperones Cdc37, Aha1, Sba1, and Sti1, which bind to Hsp90’s N-terminal domain (Röhl et al., 2013), and for co-immunoprecipitated Hsp90 (**Figure 2B**). Changing T25 to either a phosphomimetic or non-phosphorylatable form severely reduced levels of co-immunoprecipitated Hsp90 co-chaperones, possibly due to reduced Hsp90 levels or co-chaperone binding affinity. This indicates functional conservation of the role of the T25 residue with regards to co-chaperone binding.

### Hsp90 Phosphorylation Blocks Expression of Key Virulence Traits

**Figure 3:**
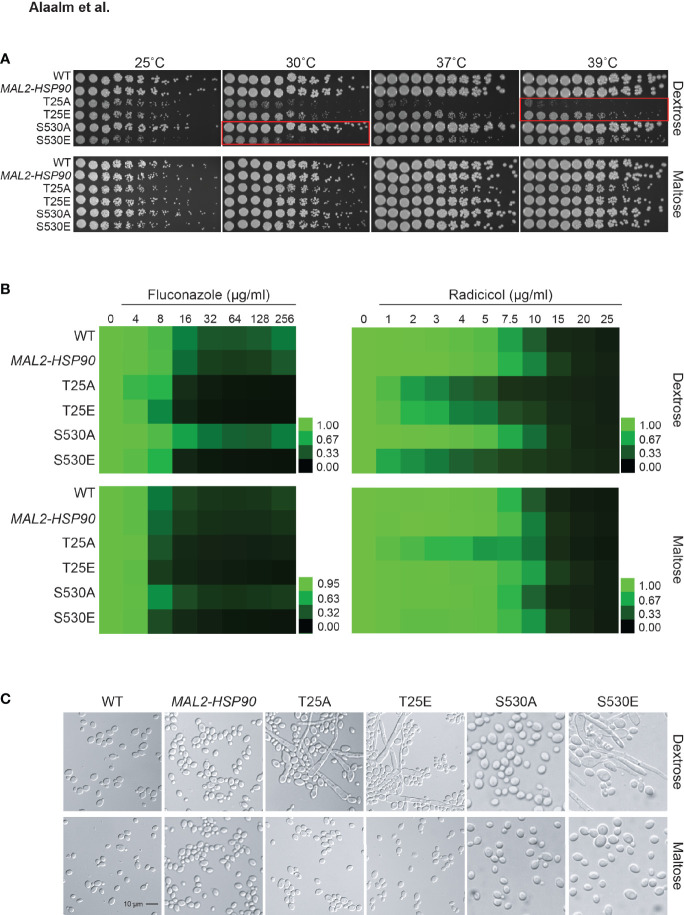
Expression of key virulence traits is contingent on Hsp90’s phosphorylation status. **(A)** Two-fold serial dilutions of the *HSP90* wild-type, the *MAL2-HSP90* promoter control strain, and *hsp90* phospho-mutants were spotted onto solid media containing either dextrose or maltose and incubated at the indicated temperatures for 48 hours. Red boxes highlighting severe phenotypes. **(B)** Wild-type and mutant cells were exposed to increasing doses of the commonly employed antifungal drug fluconazole and the Hsp90 inhibitor radicicol in media containing either dextrose (top) or maltose (bottom). Following incubation at 25˚C, cell growth was assessed, normalized to the media only control and expressed as heat map where green indicates full growth and black represents no growth. **(C)** DIC microscopic images of representative cells from each strain confirming morphogenetic changes of cells grown in dextrose (top) but not maltose (bottom). Shown is one of two biological replicates.

Mutational analysis of the *S. cerevisiae* homolog *HSP82* revealed T22 to be essential for survival of elevated temperatures (Nathan and Lindquist, 1995), a key virulence trait. To determine the importance of T25 and S530 for growth at high temperatures in *C. albicans*, *hsp90* mutants as well as the wild-type and promoter control strains were spotted onto medium containing either dextrose or maltose and exposed to different temperatures (**Figure 3A**). Although neither T25 mutant grew as robust as the wild type or the *MAL2-HSP90* control, the phosphomimetic T25E grew better than the non-phosphorylatable T25A at temperatures above 25°C. Single colonies were detectable for T25E at the highest dilutions even at 37°C and 39°C. Growth of T25A was only detectable at the lowest dilutions at elevated temperatures. In either strain, however, colonies were visibly smaller than in the control suggesting reduced growth rate due to alterations of the T25 residue. Growth of the non-phosphorylatable S530A mutant was comparable to the control strains but the phosphomimetic S530E mutant grew much less robust, especially at lower temperatures. This suggests phosphorylation of T25 and S530 plays different roles during exposure to thermal stress. Phosphorylation of T25 is required for survival of high temperatures, while phosphorylation of S530 impairs the cell’s ability to cope with lower temperatures.

Given the importance of Hsp90 in *C. albicans* antifungal drug resistance and the role of Hsp90 PTMs in susceptibility to pharmacological inhibition of Hsp90 function, we tested the phospho-mutants for susceptibility to the commonly used antifungal drug fluconazole and the Hsp90 inhibitor radicicol. Control and phospho-mutant strains were exposed to drug gradients ranging from either 0-256 µg/ml for fluconazole or 0-25 µg/ml for radicicol. Growth was quantified after 48 hours at 25°C as optical density revealing that expression of *hsp90* mutant alleles sensitizes *C. albicans* to antifungal drug treatment and Hsp90 inhibition (**Figure 3B**). Changing T25 to either a phosphomimetic or non-phosphorylatable allele or expressing the S530 phospho-allele rendered *C. albicans* susceptible to either drug. This suggests Hsp90 phosphorylation to be a determinant of drug susceptibility.

Cellular morphogenetic diversity is a fundamental element of *C. albicans’* virulence repertoire as the ability to switch between yeast and hyphae is essential for the infectious process (Saville et al., 2003). To determine how phosphorylation of T25 or S530 affects cellular morphogenesis, the wild type, the *MAL2-HSP90* control and the *hsp90* mutants were grown in standard non-filament inducing conditions in rich media at 30˚C. Microscopic examination of planktonic cells showed robust hyphal growth in both T25 mutants as well as the S530E strain in media containing dextrose, but not maltose (**Figure 3C**). To quantify these observations, we calculated the morphology index (MI) for 100 randomly selected cells for each strain (**Figure S3**). An MI > 1 is indicative of cell elongation or hyphal growth. MI scores for cells grown in dextrose or maltose differed significantly for both T25 mutants and S530E. In maltose, MIs clustered below 1, in dextrose MIs ranged from ~0.2 to ~75. Hence, both T25 mutant alleles and the phosphomimetic S530E allele cause cells to switch from yeast to hyphal growth.

### Hsp90’s Phosphorylation Status Affects Colony Appearance and Biofilm Viability

**Figure 4:**
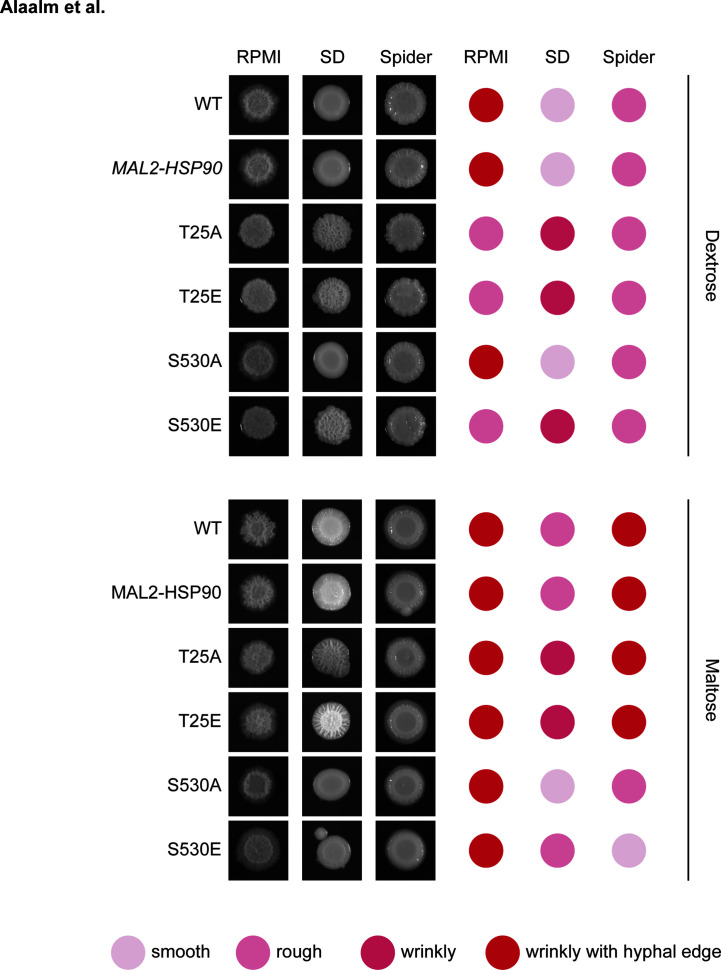
Aberrant colony morphology in response to changes in Hsp90 phosphorylation patterns. Images of colonies spotted onto RPMI, Spider or synthetic defined (SD) media containing either dextrose (top) or maltose (bottom). Colonies were scored for their appearance. The darker red the dot, the more extreme the colony phenotype. Shown is one of two replicates.

Following up on the observation that differential Hsp90 phosphorylation results in elongated cell shape, we assessed macroscopic changes to colony morphology and response to cell wall stress. To capture changes in colony morphology, all strains were grown on nutrient-limited RPMI, Spider media, which induces the yeast-to-hyphae transition, as well as synthetic defined (SD) media alone and supplemented with Calcofluor White or Congo Red (**Table S5**). Upon visual inspection of plates grown for 48 hours at 30°C, wild-type colonies were indistinguishable from the *MAL2-HSP90* control (**Figure 4**). On RPMI and SD media, colonies for both T25 mutants and the S530E mutant differed from control strains. Despite being highly filamentous on a cellular level, colonies of these three mutant strains lost their hyphal edge on RPMI. On SD, colonies for both T25 mutants as well as the S530E displayed wrinkly rather than smooth growth like the wild-type strain and the *MAL2-HSP90* control. Addition of Calcofluor White or Congo Red to SD media did not alter colony morphology (**Figure S4**). Growth on maltose restored wild type-like growth on RPMI and, to a degree, on SD media. Differences in colony morphology on Spider media appeared to be driven by the carbon source rather than a strain’s *HSP90* allele.

**Figure 5:**
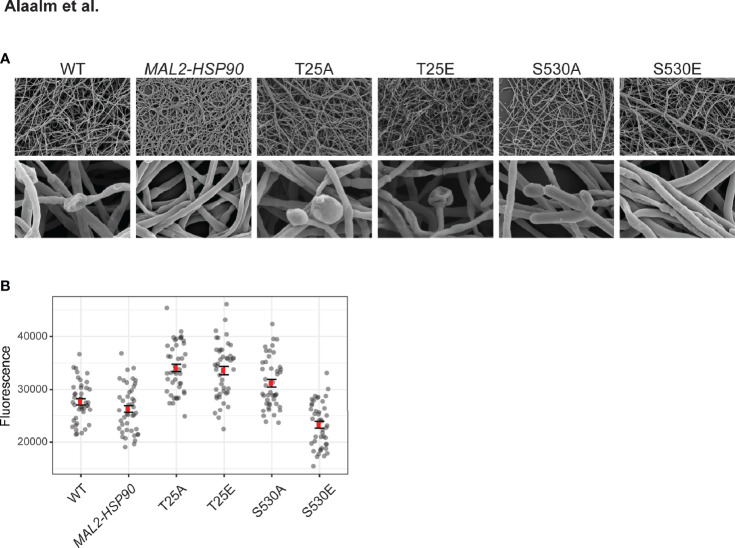
Biofilm architecture remains unaffected by Hsp90 phosphorylation. **(A)** Scanning electron microscopy images of mature biofilms at 800x (top) and 8,000x (bottom) magnification reveal biofilm architecture between the wild type, the control and the phospho-mutants to be indistinguishable upon visual inspection. **(B)** Biofilm cell viability, measured as reduction of resazurin at 590 nm, is significantly reduced in strain S530E. 44 replicates were measured per strain.

Morphogenetic diversity is a key prerequisite for the formation of *C. albicans* biofilms, which are medically relevant for two reasons. First, biofilms act as reservoirs that continuously feed the infection, and secondly they are intrinsically resistant to treatment with antifungal drugs (Lagree and Mitchell, 2017). To determine if Hsp90’s phosphorylation status affects the intricate developmental program underlying biofilm formation, cells were grown in standard biofilm-inducing conditions in RPMI (2 g/l glucose) at 37°C. Mature biofilms were imaged using scanning electron microscopy and their metabolic activity, as a measure of cell viability, was quantified employing spectrometric approaches. Microscopic analysis of biofilm architecture at 800x and 8,000x magnifications showed that control strains and phospho-mutants form structurally similar biofilms, suggesting that Hsp90’s phosphorylation status does not fundamentally affect overall biofilm development in the conditions tested (**Figure 5A**). More detailed inspection revealed that biofilm cell viability is significantly decreased in the S530E mutant (p=0.00205) relative to the *MAL2-HSP90* control (**Figure 5B**). This is in keeping with previous observations of phosphorylation of Hsp90^S530^ resulting in a loss-of-function phenotype. All other phospho-mutants, however, displayed significant increases in cell viability (T25A: p=4.01x10^-14^; T25E: p=9.94x10^-13^; S530A: p=8.68x10^-7^). While deviating from our observation of a growth defect in the T25 mutants at 37°C on YPD agar, Azadmanesh *et al*. have shown that the physical environment influences *C. albicans* gene expression and development (Azadmanesh et al., 2017).

Building on our finding that Hsp90’s phosphorylation status determines its effect on *C. albicans* cellular morphogenesis, we demonstrated that phosphorylation of S530 results in changes in colony morphology and reduced biofilm cell viability due to loss of Hsp90 function. Any manipulation of the T25 residue, however, leads to elongated cells, aberrant colony morphology and increased biofilm cell viability.

In summary, Hsp90’s phosphorylation status affects fungal virulence traits *in vitro*. Taken together, our results suggest that S530 is a novel phospho-switch. When phosphorylated, it blocks Hsp90 function and thus the expression of virulence traits, which may aid its adaptation to a commensal lifestyle while maintaining the ability to cause invasive infections. Alterations of T25, however, are important beyond phosphorylation, suggesting this residue to be a ‘switch point’, important for structural integrity of *C. albicans* Hsp90.

## Discussion

Our data indicate that phosphorylation of Hsp90^S530^ blocks expression of virulence traits while any alteration of Hsp90^T25^ resulted in a loss-of-function phenotype. This suggests that S530 is a novel *C. albicans* Hsp90 phospho-switch while T25 could act as switch point.

Although we could not detect phosphorylation of T25 in the *C. albicans* wild type or CK2 mutants, we observed that any modification of T25, be it a non-phosphorylatable alanine allele or a phosphomimetic glutamic acid residue, negatively affects co-chaperone binding, survival of high temperatures, and susceptibility to drug and Hsp90 inhibition, which is similar to what has been described in *S. cerevisiae* and mammalian cells, (Mollapour et al., 2011). Modeling surface charges revealed that modifications of T25 lead to interference with residues 373-384 in Hsp90’s middle domain. This domain is critical for client-binding as well as Hsp90 stability and function. We thus propose that T25 acts as a switch point in *C. albicans* Hsp90, although not necessarily a CK2 phosphorylation site. Switch points impact on the overall conformation and function of a protein through conformational disturbances (Rehn et al., 2020). These types of allosteric PTMs are of evolutionary significance as they expand the proteome’s complexity and facilitate dynamic responses to a large number of stimuli without the need for additional genes (Nussinov et al., 2012). The effect, however, appears to be restricted to T22 in *S. cerevisiae* and T25 in *C. albicans*, as changes in the acetylation status of the neighboring conserved *C. albicans* K30 (Li et al., 2016) or *S. cerevisiae* K27 (Robbins et al., 2012) residues did not result in loss of Hsp90 function.

Detection of Hsp90 phosphorylation sites varies greatly between different phospho-proteomic studies. Previous investigations identified between zero (Ghorai et al., 2018), nine (Cao et al., 2017) and 18 sites (Willger et al., 2015), possibly due to strain background, experimental conditions, as well as protein extraction, enrichment and mass spectrometric protocols used. Of the three sites detected here, S279 and S294 had previously been detected twice (Cao et al., 2017, Willger et al., 2015). S530 had been identified once (Willger et al., 2015). Willger et al. detected phosphorylation of Hsp90^S530^ in *C. albicans* hyphal cells (Willger et al., 2015). This finding is consistent with our experimental data showing filamentous growth in the phosphomimetic S530E mutant. While our proteomic analysis revealed S530 to be phosphorylated in the wild type, the wild-type and *MAL2-HSP90* control are phenotypically more similar to the non-phosphorylatable mutant S530A than they are to the phosphomimetic S530E mutant (**Figure 3**). This would suggest that phosphorylation of S530 is sparsely deployed and dynamic. Lack of S530 phosphorylation in the *cka2*Δ/Δ mutant encoding the α’ subunit of CK2, suggests that two of the active CK2 forms, α’ and α’_2_ββ’, are severely compromised and unable to target Hsp90. Alternatively, the blighted CK2 complex could fail to activate another kinase, which is responsible for phosphorylation of S530.

Although S530 resides in a sequence divergent region of Hsp90 (**Figure 1A**), homologous residues are almost exclusively either threonine in fungi or aspartic acid, a phosphomimetic, in fungi and animals (Figs. S1, S2). With CK2 being one of at least ten serine/threonine kinases in *S. cerevisiae* (Brinkworth et al., 2006), a close relative of *C. albicans*, it is conceivable that threonine could also be phosphorylated, suggesting mechanistic conservation. To date, only one record characterizing a homologous threonine residue exists. The *S. cerevisiae* homologous residue T533 was investigated using phosphomimetic and non-phosphorylatable alleles (Soroka et al., 2012). T533, however, does not affect survival at elevated temperatures, susceptibility to Hsp90 inhibition, or yeast growth suggesting evolutionary divergence in sequence and function. It remains to be determined if the serine residue detected in fungal plant pathogens and opportunistic pathogens of humans is of functional consequence for Hsp90 activity. At this point, the dynamic phosphorylation status of S530, which carefully orchestrates *C. albicans* virulence traits in a commensal turned pathogen yeast, and the lack of involvement in Hsp90 function of T533 in the benign yeast, *S. cerevisiae* would be in keeping with S530 to be a genetic factor that reduces severity as predicted by the trade-off hypothesis of optimal virulence theory.

In conclusion, Hsp90’s phosphorylation status and consequently structural integrity affect fungal virulence traits. CK2-mediated phosphorylation of the divergent S530 residue resulting in changes in fungal virulence traits could be further exploited as a novel therapeutic target to fight fungal infections.

## Data Availability Statement

The datasets presented in this study can be found in the github online repository: https://github.com/hobrien/DiezmannLab/blob/master/T25_analysis.Rmd.

## Author Contributions

LA, JLC, and SD conceived and designed this study. LA constructed strains, prepared samples for mass spectrometry, conducted Hsp90 stability assays and assessed co-chaperone associations. JLC conducted Hsp90 stability and drug susceptibility assays. UO performed the structural modeling analysis. RW performed Hsp90 phylogenetic analyses and ancestral character state reconstruction. CW performed *in vivo* assays. HOB analyzed growth, morphology, and biofilm data. CS assisted with mass spectrometric analyses. MB and GR performed and analyzed biofilm formation. JS modeled Hsp90 surfaces charges. SD drafted and edited the manuscript. All authors contributed to the article and approved the submitted version.

## Funding

This work was funded by MRC grant MR/L018349/1 to SD. JC was supported by the MRC GW4 ‘Biomed’ Doctoral Training Program.

## Conflict of Interest

The authors declare that the research was conducted in the absence of any commercial or financial relationships that could be construed as a potential conflict of interest.

## Publisher’s Note

All claims expressed in this article are solely those of the authors and do not necessarily represent those of their affiliated organizations, or those of the publisher, the editors and the reviewers. Any product that may be evaluated in this article, or claim that may be made by its manufacturer, is not guaranteed or endorsed by the publisher.
